# Simulation and Analysis of Mie-Scattering Lidar-Measuring Atmospheric Turbulence Profile

**DOI:** 10.3390/s22062333

**Published:** 2022-03-17

**Authors:** Yuqing Lu, Jiandong Mao, Yingnan Zhang, Hu Zhao, Chunyan Zhou, Xin Gong, Qiang Wang, Yi Zhang

**Affiliations:** 1 School of Electrical and Information Engineering, North Minzu University, North Wenchang Road, Yinchuan 750021, China; 20197209@stu.nun.edu.cn (Y.L.); 20207206@stu.nun.edu.cn (Y.Z.); 1996013@nmu.edu.cn (H.Z.); 2007050@nmu.edu.cn (C.Z.); 2017004@nmu.edu.cn (X.G.); 2013057@nmu.edu.cn (Q.W.); 2013091@nmu.edu.cn (Y.Z.); 2 Key Laboratory of Atmospheric Environment Remote Sensing of Ningxia Province, North Wenchang Road, Yinchuan 750021, China

**Keywords:** Mie lidar, atmospheric turbulence, residual turbulent scintillation, scintillation index, atmospheric refractive index structure constant

## Abstract

Based on the residual turbulent scintillation theory, the Mie-scattering lidar can measure the intensity of atmospheric turbulence by detecting the light intensity scintillation index of the laser return signal. In order to evaluate and optimize the reliability of the Mie-scattering lidar system for detecting atmospheric turbulence, the appropriate parameters of the Mie-scattering lidar system are selected and optimized using the residual turbulent scintillation theory. Then, the Fourier transform method is employed to perform the numerical simulation of the phase screen of the laser light intensity transformation on the vertical transmission path of atmospheric turbulence. The phase screen simulation, low-frequency optimization, and scintillation index calculation methods are provided in detail, respectively. Based on the phase distribution of the laser beam, the scintillation index is obtained. Through the relationship between the scintillation index and the atmospheric turbulent refractive index structure constant, the atmospheric turbulence profile is inverted. The simulation results show that the atmospheric refractive index structure constant profile obtained by the iterative method is consistent with the input HV_5/7_ model below 6500 m, which has great guiding significance to carry out actual experiments to measure atmospheric turbulence using the Mie lidar.

## 1. Introduction

Atmospheric turbulence is a kind of irregular vortex motion and is generated mainly due to three reasons: wind shear caused by the drag of the air on the Earth’s surface, thermal convection caused by surface heat radiation, and fluctuation in the temperature and velocity fields due to heat-releasing phase transition processes (deposition and crystallization) [1]. The physical properties of turbulence, such as pressure, speed, and temperature, are random. The atmospheric turbulence effect will cause a series of optical phenomena, including light intensity scintillation, beam drift, beam expansion, and arrival angle fluctuation [2]. Among them, the light intensity scintillation will cause the loss of received power, thereby reducing the signal-to-noise ratio of the system. In fact, atmospheric turbulence has a certain impact on the study of adaptive optics [3,4], atmospheric laser communication [5], astronomical site selection, and astronomical observation image analysis [6]. However, there is no complete model to fully describe the impact of atmospheric turbulence [7].

The Navier–Stokes (NS) equation is the basis for many atmospheric turbulence calculations. According to the different processing scales of atmospheric turbulence, it is divided into direct numerical simulation (DNS), Reynolds averaged Navier–Stokes (RANS), and large eddy simulation (LES). Among them, the direct numerical simulation method does not need any turbulence model, artificial assumptions, or empirical constants, and in principle can solve all atmospheric turbulence problems [8]. Therefore, numerical modeling is usually used as one of the main research methods to study atmospheric turbulence. Numerical modeling usually includes the Fourier transform method (FFT), Zernike polynomial higher-order method (Zernike), fractal method (Fractal), and some hybrid methods, among which the Fourier transform method has been applied widely in the turbulent phase screen simulation due to its fast calculation advantage, especially in the occasions with high real-time requirements, such as space optical communication and atmospheric laser transmission. However, the Fourier transform method has serious defects in the low-frequency part, which makes the phase screen have a large error in characterizing large-scale turbulent eddies [9]. Therefore, some research has been performed to overcome the defect. In 1992, Lane et al. proposed a multi-order harmonic method to improve the low-frequency compensation, simulation accuracy, and calculation speed [10]. In 2008 and 2009, Qian et al. studied the distribution of the phase screen and the selection of calculation parameters [11,12]. In 2013, Herman and Strungala proposed a method of adding sub-harmonics to perform low-frequency compensation on the phase screen generated by the FFT method [13]. In 2013, Charnotskii et al. proposed a sparse spectral model, which superimposed the dense sampling of the low-frequency part and the sparse sampling of the high-frequency part, and achieved good results [14]. In 2017, Feng et al. proposed a phase screen simulation of atmospheric turbulence based on wavelet analysis and found that it was in good agreement with the von Karman spectrum and the computational complexity was reduced [15]. In 2019 and 2020, Zhang et al. proposed an optimization algorithm for sparsely resampling the low-frequency region with the help of the gravitational search algorithm to form a low-frequency compensation screen [16,17].

Moreover, in using the scintillation index to study atmospheric turbulence, some research has been carried out. In 2006, Rao et al. conducted a simulation study on the scintillation index of Gaussian beams in atmospheric turbulence and verified that the scintillation index increases with the distance from the optical axis [18]. In 2017, Han et al. used the step Fourier method to simulate the two-way transmission of the laser and analyzed the backward enhancement effect of the plane mirror and the angle of reflection on the scintillation index by calculating the scintillation index of the retroreflection spot [19]. In 2018, Li et al. simulated the scintillation index of plane waves propagating in atmospheric turbulence with the non-Kolmogorov spectrum and compared the variation of the scintillation index of the Kolmogorov spectrum and the non-Kolmogorov spectrum alone on the turbulent path [20].

An active optical remote sensing detection method is important for atmospheric turbulence measurement. The differential image movement measurement method (DIM) uses artificial light sources, which makes the measurement susceptible to light sources and has low resolution [21]. The Doppler wind profiler (MST) measurement method has high sensitivity and versatility but is easily affected by weather and has a limited measurement range [22]. As an important active remote sensing method, lidar has the characteristics of high resolution and high precision and is a reliable and effective means to detect atmospheric parameters. The backscattering amplification effect of the lidar (BSA) measurement method has high spatial, temporal, and resolution features as well as good real-time performance, and is not affected by time integration [23], while the scintillation lidar (DCIM) has accurate detection and low measurement cost [24]. In this paper, considering the advantages and disadvantages of various measurement methods, a Mie-scattering lidar system is selected for atmospheric turbulence measurement. According to the residual turbulent scintillation (RTS) theory [25], it was improved by adding small aperture diaphragms with appropriate apertures [26]. The atmospheric turbulence profile is obtained by inversion using the light intensity information at different heights of the return signal of the Mie-scattering lidar system.

In this paper, based on the RTS theory, the Mie-scattering lidar is selected to measure the intensity of atmospheric turbulence and the numerical simulation is carried out by using the phase screen simulation. In the simulation, the improved fast Fourier transform method with sub-harmonics is used to simulate the transmission of laser beams in atmospheric turbulence. After atmospheric extinction and backscattering, telescope imaging spots at different heights from the Mie-scattering lidar return signal are obtained. By analyzing the imaging spot, the scintillation change of its light intensity can be obtained. Moreover, by understanding the influence of specific measurement parameters on atmospheric turbulence detection, the reliability of its detection performance can be judged and follow-up guidance for system evaluation and optimization can be provided.

## 2. Numerical Simulation Model

### 2.1. Mie-Scattering Lidar

**Figure 1:**
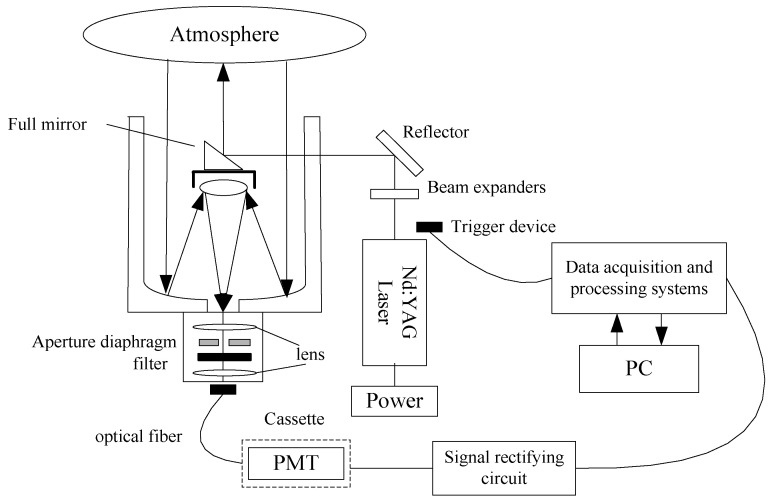
The structure diagram of the Mie lidar system.

The schematic diagram of the Mie-scattering lidar is shown in Figure 1. The collimated laser beam emitted by the transmitting system is expanded by the beam expander and then transmitted to the atmosphere [27]. Through the combined action of atmospheric Fresnel diffraction, atmospheric extinction and atmospheric turbulence [28], the backscattered return signal is received by the receiving system and then passes through the spectroscopic system. The return signals of each wavelength are separated and the Mie-scattering signal with a wavelength of 532 nm is selected for our goal. Finally, the return signal is detected, amplified, and displayed by the signal acquisition system. Table 1 shows the system parameters of the Mie-scattering lidar [29].

The improved Mie-scattering lidar system is mainly based on the RTS theory, and a suitable aperture diaphragm is added to the original Mie-scattering lidar system to meet the needs of atmospheric turbulence detection. In RTS theory [25], when light propagates in the atmosphere, if the radius of the scatterer is smaller than the spatial correlation scale $l_{I}$ ($l_{I}=\sqrt{\lambda L}$, where λ is the wavelength of the incident laser light, *L* is the laser detection distance), for any large receiving aperture *D*, the backscattered light intensity signal can reduce the aperture averaging effect, and there will be some light intensity fluctuations.

The following four conditions are required to be satisfied: (1) the single scattering is approximate and the aerosol particle size is uniform; then, the laser detection distance *L*, the average radius of aerosol particles $\bar{a}$, and the incident light wavelength λ, satisfy $L\gg {\bar{a}}^{2}/\lambda$; (2) atmospheric optical thickness τ≪1; (3) the radial light intensity correlation scale $l_{I∥}$ ($l_{I∥}\approx L$) is much larger than the scatterer scale cτ/2 (where c is the speed of light, τ is the pulse width), and the scatterer scale is much larger than the wavelength, that is, L≫cτ/2≫λ; and (4) under weak fluctuations ($\sigma_{I}{}^{2}\ll 0.6$), the telescope receiving aperture *D*, the turbulent coherence length of plane waves $\rho_{0}$,${\;l}_{I}$ and $\rho_{\mathrm{cv}}$ satisfy $D\gg \rho_{0}\gg l_{I}\gg \rho_{\mathrm{cv}}$ (where $\sigma_{I}{}^{2}$ is the logarithmic light intensity fluctuation variance; $\rho_{\mathrm{cv}}=\frac{2L}{ka_{\mathrm{ef}}}$, where $\rho_{\mathrm{cv}}$ is the light intensity coherence length derived from the Van–Cittert–Zernike theory, $a_{\mathrm{ef}}$ is the effective size of the beam from the light source *L*, and k is the wave number, k=2π/λ). The lidar can be distinguished by two characteristic scales, namely, aerosol spot scale, $l_{a}=\lambda F/D$, and turbulence-related scale, $l_{t}=Fl_{I}/L$, where *F* is the focal length of the telescope. Note that $l_{a}/l_{t}=D/\sqrt{\lambda L}$, so it generally satisfied $l_{t}\gg l_{a}$ for most large-aperture receiving systems ($D\gg \sqrt{\lambda L}$).

Because the spatial scales $l_{a}$ and $l_{t}$ of the scintillation spot are completely different, three different expressions for the fluctuation of the backscattered light intensity can be obtained by changing the diameter of the telescope’s field of view diaphragm $d_{0}$:

(1) $$\sigma_{p}{}^{2}=1+2\sigma_{I}{}^{2},\;\;\;\;\;\;\;\;\;d_{0}\ll l_{a}$$

(2) $$\sigma_{p}{}^{2}=\sigma_{I}{}^{2},\;\;\;\;\;\;\;\;\;l_{a}\ll d_{0}\ll l_{t}$$

(3) $$\sigma_{p}{}^{2}=O(\sigma_{I}{}^{2}/{\left(\frac{d_{0}}{F\sqrt{\lambda /L}}\right)}^{7/3}),\;l_{t}\ll d_{0}$$

where, $\sigma_{p}{}^{2}$ is the variance of the intensity fluctuation of the backscattered light passing through the aperture; $\sigma_{I}{}^{2}$ is the variance of the intensity fluctuation of the probe beam. Equation (1) shows that the variance of light intensity fluctuations includes aerosol speckle fluctuations and turbulence fluctuations. Equation (2) shows that the diaphragm smooths the aerosol speckle fluctuations but includes turbulence fluctuations. Equation (3) shows that aerosol speckle fluctuations and turbulence fluctuations are smoothed by the diaphragm, and the total received energy does not fluctuate.

**Figure 2:**
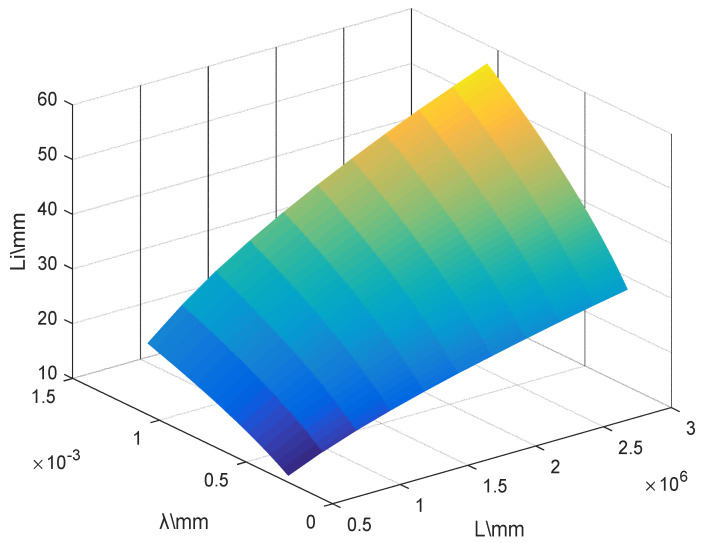
Spatial correlation scale of light intensity fluctuation $l_{I}$.

In this paper, the selected telescope aperture *D* is 10 inches (254 mm), the repetition frequency *f* is 10 Hz, and the adjustable focal length *F* is 2500 mm. Considering the experimental operability and the actual laboratory conditions, the selected field of view of the telescope is $d_{0}\geq 0.2\;\mathrm{mm}$. According to the RTS theory, the spatial correlation scale $l_{I}$ ($l_{I}=\sqrt{\lambda L}$) of the light intensity fluctuation is calculated as shown in Figure 2. The spatial correlation scale $l_{I}$ increases with the increase in the detection distance and incident wavelength. When the detection distance is the maximum value of 3 km and the incident laser wavelength takes the maximum value of 1064 mm, $l_{I}$ gets the maximum value of 57.5 mm, which obviously satisfies $D\gg l_{I}$. In addition, according to our laboratory’s long-term measurement results of aerosol particle spectral distribution in the Yinchuan area, China, it can be known that the aerosol particle size $\bar{a}=0.9\;\mu m$. Yinchuan area is located in the junction of the four deserts, namely Badain Jaran, Ulan Buhe, Tengger, and Mu Us deserts, combined with the typical temperate continental semi-arid climate, the aerosol particle size in spring is slightly larger than the average; obviously, all meet the condition $a_{r}\ll l_{I}$. Therefore, the improved Mie-scattering lidar system can satisfy the RTS theory. By changing the diameter of the field of view diaphragm, the information characterizing atmospheric turbulence can be obtained from the return signal.

**Figure 3:**
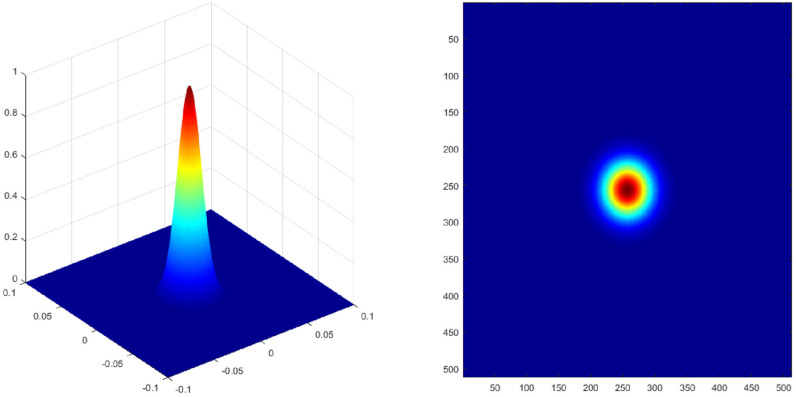
The three-dimensional (**left**) and two-dimensional (**right**) schematic diagrams of the emitted beam intensity distribution of the Mie lidar system at *z* = 0.

Gaussian beam is one of the most commonly used models in lasers. In fact, the light intensity of a Gaussian beam in a plane is perpendicular to the direction of propagation. A Gaussian beam in a homogeneous medium is expressed as:

(4) $$E\left(x,y,z\right)\;=\;E_{0}\frac{\omega_{0}}{\omega \left(z\right)}\exp\{-[\frac{r^{2}}{\omega {\left(z\right)}^{2}}+i\frac{k}{2R\left(z\right)}]\}$$

(5) $$I\left(x,y,z\right)\;=\;E_{0}{}^{2}\frac{\omega_{0}{}^{2}}{\omega {\left(z\right)}^{2}}\exp\{-[\frac{2r^{2}}{\omega {\left(z\right)}^{2}}]\}$$

where, $\omega \left(z\right)$ is the beam radius (i.e., $\omega \left(z\right)\;=\;\omega_{0}\sqrt{1+z^{2}/z_{0}^{2}}$), $\omega_{0}$ is the minimum value of *ω*(*z*) at *z* = 0, which is called the light waist radius, *r* is the distance between a point in space (*x*, *y*, *z*) and the light source; and *k* is the wave number (i.e., k=2π/λ). Figure 3 shows the light intensity distribution of the Gaussian beam with a beam waist radius of 20 mm, wavelength λ=532 nm, and z=0. It can be seen that the light spot is a regular circle, and the light spot is at the center.

### 2.2. Numerical Simulation of Gaussian Beam Transmission in Atmosphere

**Figure 4:**
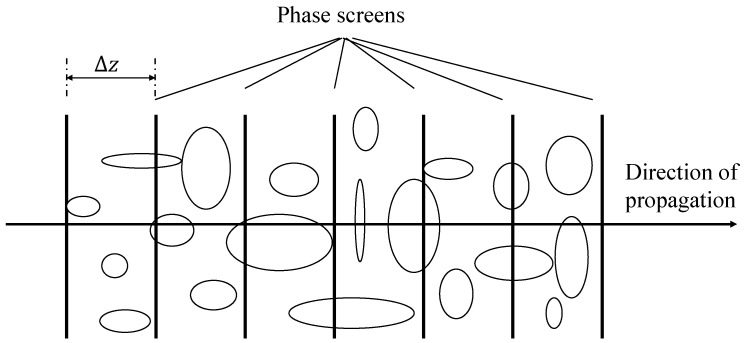
Phase screen model of light transmission in atmospheric turbulence.

When the laser is transmitted in the atmosphere, it is subjected to the dual action of the turbid atmosphere and continuous turbulence and then diffracted in the atmospheric space, resulting in atmospheric scattering and absorption and a series of turbulent effects caused by turbulent refraction. In the phase screen model, the light propagation in atmospheric turbulence mainly starts from the light propagation equation. Assuming that the transmission path of the light beam is composed of several thin phase screens distributed in a vacuum environment, and when the phase change caused by the refractive index of the medium is particularly small, the process of light propagation in a vacuum environment and the phase modulation of the medium can be regarded as completely independent of each other and completed simultaneously [1]. That is, the multi-layer phase screen model of light propagation in a continuous random medium is shown in Figure 4, in which the circles or ellipses represent random media in the atmosphere, such as atmospheric molecules, aerosols, etc. The phase disturbance is firstly generated by a thin phase screen with negligible thickness. After propagating through the vacuum of distance Δz, the phase disturbance of the previous phase screen is superimposed on this phase screen to repeat the phase modulation, with the above process repeating until the end of the transmission.

Assuming that the light travels along the z direction, the light field can be expressed as $E=ue^{ikz}$. When the laser passes through the atmospheric turbulence with a refractive index of $n=1+n_{1}$, under the condition of paraxial approximation, the light propagation satisfies the parabolic equation:

(6) $$2ik\frac{\partial u}{\partial z}+\frac{\partial^{2}u}{\partial^{2}x}+\frac{\partial^{2}u}{\partial^{2}y}+2k^{2}n_{1}u=0$$

where, k=2π/λ, λ is the wavelength of the light wave, k is the wave number, and $n_{1}$ is the refractive index fluctuation.

Let the transmission distance *L* of the light wave be divided into $N_{z}$ segments according to Δz equal intervals, so the head and tail coordinates of the *i*-th segment are $z_{i-1}$ and $z_{i}$, respectively, and the *i*-th phase screen is set at the endpoint $z_{i}$ of each segment. For each phase screen, the grid spacing of Δx is equally divided into N×N grids, and the above formula is solved by the step-by-step method. It can be known that the field distribution of the *I* + 1-th phase screen is:

(7) $$u_{i+1}=F^{-1}[F\left\{u_{i}\exp\left(ikn_{1}\Delta z\right)\right\}\exp\left(-i\frac{\kappa_{x}^{2}+\kappa_{y}^{2}}{2k}\Delta z_{i}\right)]$$

where, $F^{-1}$ and F are the inverse Fourier transform and Fourier transform, $u_{i}$ is the field distribution of the *i*-th phase screen, $\Delta z_{i}=z_{i+1}-z_{i}$ is the distance between the phase screen, and $\kappa_{x}$ and $\kappa_{y}$ are the phase space wave number with the unit of rad/m, which are related to the scale of turbulence.

The most important problem in the numerical simulation method of light propagation is to construct the appropriate phase screen, so that it can accurately reflect the change in the atmospheric turbulent refractive index as much as possible [30], that is, to choose the appropriate refractive index fluctuation $n_{1}$. We use the spatial spectrum model of atmospheric turbulence to obtain a random field of the phase space, and then use Fourier transform to get the spatial distribution of the two-dimensional phase. The specific process is as follows: generate a complex Gaussian random number matrix $a_{R}$, select an atmospheric turbulence power density spectral function $\phi_{n}$ to filter it to obtain a random field in the complex space, and perform inverse Fourier transform on the discrete complex random field to obtain the real phase distribution in space.

Atmospheric turbulence refractive index spectral function $\phi_{n}$ and atmospheric turbulence phase spectral function $\phi_{\phi }$ using Kolmogorov spectrum [2] are written by:

(8) $$\phi_{n}\left(\kappa \right)\;=\;0.033{\;*\;C}_{n}{}^{2}{(h)\;*\;\kappa }^{-11/3}\;\;\;\;\;\;\;\;\;1/L_{0}\leq \kappa \leq 1/l_{0}$$

(9) $$\phi_{\phi }\left(\kappa \right)\;=\;2\pi \;*\;k^{2}\;*\;\Delta z\;*\;\phi_{n}\left(\kappa \right)$$

where, $C_{n}{}^{2}$ is the atmospheric refractive index structural constant when the laser travels along the horizontal path, and the unit is $m^{-2/3}$; κ is the spatial frequency; $L_{0}$ is the turbulent outer scale, and $l_{0}$ is the turbulent flow inner scale, which determines the largest and small eddies in the turbulent eddy.

The complex space random field obtained after filtering the complex Gaussian random number matrix $a_{R}$ is given by:

(10) $$\tilde{S}(\kappa_{x},\;\kappa_{y})\;=\;a_{R}\sqrt{\phi_{\phi }\left(\kappa_{x},\kappa_{y}\right)}$$

(11) $$a_{R}\;=\;A_{R}+iB_{R}$$

where, $A_{R}$ and $\;B_{R}$ are Gaussian random numbers whose real and imaginary parts have a mean of 0 and a variance of 1. The real and imaginary parts of the phase field obtained by Equation (10) are independent of each other, and both satisfy the spatial–spectral distribution.

**Figure 5:**
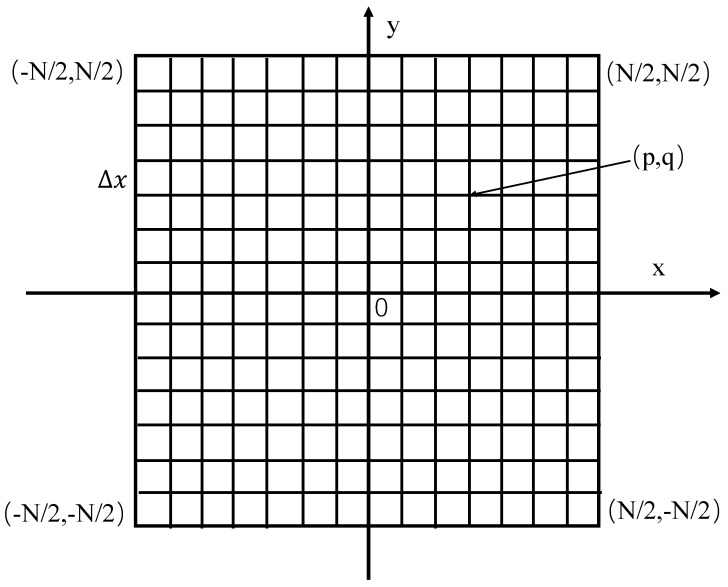
Schematic diagram of phase screen grid simulation.

From the above, the field distribution in the continuous space can be obtained. However, the numerical simulation needs to generate a discrete random field, that is, it needs to perform discrete Fourier transform on it. Taking a phase screen as an example, as shown in Figure 5, the square phase screen with side length *L* is divided into *N* equal parts to form N×N square grids with a spacing of ∆x. Then, the discrete Fourier transform of Equation (10) can be obtained:

(12) $$\tilde{S}(p∆k,\;q∆k)\;=\;a_{R}\sqrt{\phi_{\phi }(p∆k,\;q∆k)}/∆k$$

where, ∆k is the wavenumber interval of the phase space, according to the sampling theorem, $∆k=2\pi /\left(N∆x\right)$.

In order to obtain the phase distribution in real space, the inverse Fourier transform of Equation (12) can be given by:

(13) $$S_{hig}(p∆x,\;q∆x)=\frac{1}{N^{2}}\overset{N-1}{\underset{m=0}{\sum }}\overset{N-1}{\underset{n=0}{\sum }}\tilde{S}(p∆k,\;q∆k)\exp\left[-2\pi i\left(mp+nq\right)/N\right]$$

### 2.3. Low-Frequency Sampling Optimization

For the simulated turbulent phase screen generated by the Fourier transform method, the range of the minimum wave number interval ∆k of the phase screen does not include $\left(-∆\kappa_{x}/2,\;∆\kappa_{x}/2\right)$ and $\left(-∆\kappa_{y}/2,\;∆\kappa_{y}/2\right)$, so it has the disadvantage of insufficient low frequency [31]. We use the method of adding sub-harmonics to perform low-frequency compensation on the phase screen by the Fourier transform method. In essence, the high-frequency phase screen simulated by the FFT method is interpolated and fitted to improve the statistical characteristics of low frequency. The specific process is as follows: firstly, divide the square area surrounded by the first sampling point of the low-frequency part limited to the high-frequency part into nine areas with equal area; then, set the sampling points contained in the area (including the edge part) to zero; and finally, take eight small areas outside the center as new sampling points to form a p-order (p≥1) harmonic network.

**Figure 6:**
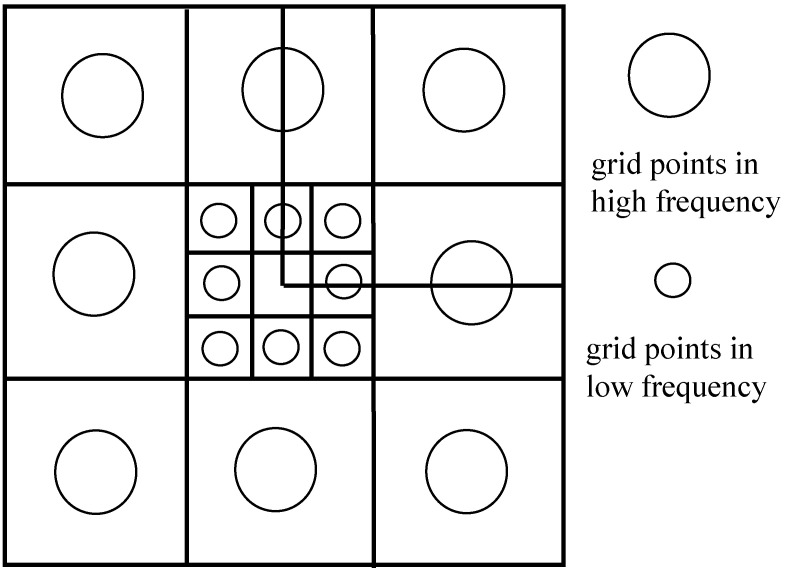
Schematic diagram of sub-harmonic compensation.

As shown in Figure 6, the sampling interval of the *p*-th harmonic network becomes $∆f_{p}=∆k/3^{p}$, that is, the original high-frequency sampling area is replaced by $3^{-2p}$ low-frequency sampling small areas. According to the Kolmogorov spectrum, the subharmonic low-frequency compensation screen can be expressed as:

(14) $$S_{low}(p^{\prime }∆x,\;q^{\prime }∆x)\;=\;\overset{N_{P}}{\underset{P=1}{\sum }}\overset{1}{\underset{m^{\prime }=0}{\sum }}\overset{1}{\underset{n^{\prime }=0}{\sum }}\tilde{S}(p^{\prime }∆x,\;q^{\prime }∆x)\exp\left[-2\pi i3^{-P}\left(\frac{m^{\prime }p}{N}+\frac{n^{\prime }q}{N}\right)\right]$$

(15) $${\tilde{S}}_{low}(p^{\prime }∆x,\;q^{\prime }∆x)=a_{R}{}^{\prime }\sqrt{\phi_{\phi }(p^{\prime }∆x,\;q^{\prime }∆x)}/∆k$$

Therefore, the phase screen expression after sub-harmonic compensation is written by:

(16) $$S\left(p∆x,\;q∆x\right)\;=\;S_{hig}(p∆x,\;q∆x)+S_{low}(p^{\prime }∆x,\;q^{\prime }∆x)$$

The phase structure function is a quantity that describes the statistical properties of the atmospheric turbulence phase screen, and the accuracy of the simulation is generally judged by a large number of phase screens. The phase structure function is defined by [32]:

(17) $$D_{\emptyset }\left(\vec{r}\right)\;=\;\langle {\left[\emptyset \left(\vec{\rho }+\vec{r}\right)-\emptyset \left(\vec{\rho }\right)\right]}^{2}\rangle \;=\;2\left(B_{\emptyset }\left(0\right)-B_{\emptyset }\left(\vec{r}\right)\right)$$

When the laser is transmitted in the atmospheric turbulence whose spectral model takes the Kolmogorov spectrum, the theoretical expression of the structure function is given by [32]:

(18) $$D_{\emptyset }\left(\vec{r}\right)\;=\;2.91k^{2}{\left|\vec{r}\right|}^{5/3}\int_{0}^{1}{\left(\left(l-∆z\right)/l\right)}^{5/3}\cdot C_{n}^{2}\left(s\right)ds$$

For the inner scale, taking infinity ($\kappa_{0}\to \infty$) and infinitely small, there are:

(19) $$D_{\emptyset }\left(\vec{r}\right)\;=\;6.88{\left(\left|\vec{r}\right|/r_{0}\right)}^{5/3}$$

(20) $$D_{\emptyset }\left(\vec{r}\right)\;=\;6.16r_{0}{}^{5/3}\left[\frac{3}{5}{\left(\frac{L_{0}}{2\pi }\right)}^{5/3}-{Κ}_{5/6}\frac{{\left(\frac{rL_{0}}{4\pi }\right)}^{5/6}}{\Gamma \left(11/6\right)}(\frac{2\pi r}{L_{0}})\right]$$

where ${Κ}_{5/6}$ is the modified Bessel function of the third kind, and $\Gamma \left(11/6\right)$ is the Gamma function.

From a statistical point of view, when giving a large number of random phase screen samples, its structure function will gradually converge to its expected structure function. In order to reduce the calculation amount, the expected structure function can be calculated directly [17]. The specific process is as follows: perform inverse Fourier transform on the phase spectral function $\phi_{\phi }\left(\vec{\kappa_{r}}\right)$ to obtain the auto correlation function $B_{\emptyset }\left(\vec{r}\right)=\iint_{-\infty }^{+\infty }\phi_{\phi }\left(\vec{\kappa_{r}}\right)e^{i\vec{r}\cdot \vec{\kappa_{r}}}d\vec{\kappa_{r}}$, and then substituting it into Equation (17) can get its structure function. The auto correlation functions of the high-frequency phase screen and low-frequency compensation can be written by:

(21) $$B_{\emptyset }\left(p,\;q\right)=\sum_{m=0}^{N-1}\sum_{n=0}^{N-1}f^{2}\left(p,\;q\right)\;exp\left[-2\pi i\left(mp+nq\right)/N\right]$$

(22) $$B_{\emptyset }\left(p^{\prime },\;q^{\prime }\right)=\sum_{p=1}^{N_{p}}\sum_{m=0}^{N-1}\sum_{n=0}^{N-1}f^{2}\left(p^{\prime },\;q^{\prime }\right)\;exp\left[-2\pi i3^{-p}\left(mp+nq\right)/N\right]$$

where, $f\left(p,\;q\right)$ and $f\left(p^{\prime },\;q^{\prime }\right)$ are the spatial filter functions, $f\left(p,\;q\right)\;=\;\frac{2\pi }{\Delta x\cdot N}\sqrt{0.00058}r_{0}{}^{-5/6}{\left({\left(\frac{p}{\Delta x\cdot N}\right)}^{2}+{\left(\frac{q}{\Delta x\cdot N}\right)}^{2}\right)}^{-11/12}$, $f\left(p^{\prime },\;q^{\prime }\right)\;=\;\frac{2\pi 3^{-p}}{\Delta x\cdot N}\sqrt{0.00058}r_{0}{}^{-5/6}{\left({\left(\frac{p^{\prime }\cdot 3^{-p}}{\Delta x\cdot N}\right)}^{2}+{\left(\frac{q^{\prime }\cdot 3^{-p}}{\Delta x\cdot N}\right)}^{2}\right)}^{-11/12}$, and $r_{0}$ is the atmospheric coherence length, which is taken as 0.185.

### 2.4. Scintillation Index of Laser Return Signal

For the RTS theory, the light intensity change of the lidar return spot is the research focus. Under weak turbulence conditions, the scintillation index $\beta_{I}^{2}$ is one of the commonly used physical quantities to describe atmospheric turbulence. $\beta_{I}^{2}$ represents the normalized light intensity fluctuation difference, which is defined as:

(23) $$\beta_{I}^{2}\left(\rho ,\;L\right)\;=\;\frac{\langle I^{2}\rangle -{\langle I\rangle }^{2}}{{\langle I\rangle }^{2}}$$

where, < > represents the ensemble mean of the light intensity, and ρ represents the radial distance from the optical axis of the Gaussian beam.

It is known that the Gaussian beam will produce spot drift after transmission in atmospheric turbulence. In order to obtain its scintillation index $\beta_{I}^{2}\left(\rho_{i},\;L_{i}\right)$ through several light spots with a transmission distance of $L_{i}$, one need to judge the drift distance $\rho_{i}$ of the center of mass of the light spot [33]. The spot centroid is defined as:

(24) $$\rho_{c}=\iint \rho I\left(\rho \right)d\rho /\iint I\left(\rho \right)d\rho$$

Usually, its first moment is used to represent the coordinates of the centroid of the spot, namely [1]:

(25) $$x_{c}=\iint xI\left(x,\;y\right)dxdy/\iint I\left(x,\;y\right)dxdy$$

(26) $$y_{c}=\iint yI\left(x,\;y\right)dxdy/\iint I\left(x,\;y\right)dxdy$$

In the simulation, ten light spots with a transmission distance of $\rho_{i}$ are taken. For each light spot, if $\rho_{i}\leq 15∆x$, the center of mass of the drifted light spot is selected as the center of the circle. In addition to this, take 15∆x as the radius to make a circle, and count the light intensity of points in the circle. If $\rho_{i}>15∆x$, the center of mass of the spot after the drift is also taken as the circle center. Then, $\rho_{i}-1.5∆x$ and $\rho_{i}+1.5∆x$ are used as the radii to make a circle, respectively. Through counting the light intensity of *N* nodes in the ring, the scintillation index can be calculated by the definition formula $\beta_{I}^{2}\left(\rho_{i},\;L_{i}\right)=\frac{{\langle I^{2}\rangle }_{i}-{\langle I\rangle }^{2}{}_{i}}{{\langle I\rangle }^{2}{}_{i}}$. Finally, ten statistical averages are performed to obtain the final scintillation index at a certain distance.

For a Gaussian beam, according to the Rytov theory, the spot scintillation index of the beam is defined as:

(27) $$\beta_{I}^{2}\left(\rho ,\;L\right)=4\times 2.17k^{7/6}Re\left\{\{{\left[i\gamma \left(L-z\right)\right]}^{5/6}-{\left[\gamma_{i}\left(L-z\right)\right]}^{5/6}\}C_{n}^{2}\left(z\right)dz\right\}$$

where, k = 2π/λ, $\gamma \;=\;\left(1+iaz\right)/\left(1+iaL\right)\;=\;\gamma_{r}-i\gamma_{i}$, $a\;=\;\lambda /\pi W_{0}^{2}+i/R_{0}$.

### 2.5. Inversion of Turbulent Profile

The refractive index structure constant $C_{n}{}^{2}$ can directly describe the fluctuation of the refractive index and is one of the most intuitive physical quantities to measure the intensity of atmospheric optical turbulence. The atmospheric turbulence intensity profile is the variation of atmospheric refractive index structure constant $C_{n}{}^{2}$ with height [33]. In this paper, the refractive index structure constant $C_{n}{}^{2}$ can be calculated from the correlation relationship between the known spherical wave scintillation index $\;\beta_{I,S}{}^{2}$ and the atmospheric refractive structure constant $\;C_{n}{}^{2}$ in the horizontal and vertical directions, so as to obtain the atmospheric turbulence intensity profile.

Kolmogorov believes that atmospheric turbulence is composed of turbulent eddies with great difference and different scales. Under the large Reynolds number *R*e (Re=UL∕ν, where U is the characteristic velocity of the fluid, L is the overall characteristic scale of the fluid, and *ν* is the molecular kinematic viscosity coefficient), the turbulent eddies of different scales coexist. After the cascade process, the small-scale turbulence finally reaches a statistical equilibrium process, that is, local isotropic turbulence [2]. The three-dimensional power spectrum function of the locally isotropic turbulent refractive index fluctuation is expressed as:

(28) $$\phi_{n}\left(\kappa \right)\;=\;0.033{\;*\;C}_{n}{}^{2}{\;*\;\kappa }^{-11/3}\;*\;f\left({\kappa l}_{0}\right)$$

where, $\phi_{n}\left(\kappa \right)$ is the atmospheric turbulence power spectrum function, *κ* is the spatial frequency, $l_{0}$ is the turbulent inner scale, that is, the scale that determines the smallest vortex in the turbulent vortex, and $f\left(\kappa l_{0}\right)$ is the inner scale correction model factor.

The Kolmogorov atmospheric turbulence power spectrum is an ideal power spectrum model, which does not consider the inner scale effect of turbulence. It considers that the outer scale *L*_0_ is infinite and the inner scale *l*_o_ is zero [34]. At this time, $f\left(\kappa l_{0}\right)\;=\;1$. So the three-dimensional power spectrum function is expressed as:

(29) $$\phi_{n}\left(\kappa \right)\;=\;0.033\;*\;C_{n}{}^{2}\;*\;\kappa^{-11/3}\;$$

Under the condition of weak fluctuation ($\beta_{I}{}^{2}<1$), the scintillation index satisfies:

(30) $$\;\;\beta_{I}{}^{2}\;=\;\exp\left(4\sigma_{\chi }^{2}\right)-1\;\approx \;4\sigma_{\chi }^{2}\;$$

(31) $$\sigma_{\chi }^{2}\;=\;{\left(2\pi k\right)}^{2}\int_{0}^{L}dz\int_{0}^{\infty }sin^{2}\left[P\left(\gamma ,\kappa ,z\right)\right]\phi_{n}\left(\kappa \right){|}_{z}\kappa d\kappa$$

where, $\sigma_{\chi }^{2}$ is the logarithmic amplitude fluctuation variance, k = 2π/λ is the wave number, and κ is the spatial frequency.

In the vertical direction, according to the Kolmogorov spectrum, the relationship between the spherical wave scintillation index $\beta_{I,S}{}^{2}$ and the refractive index structure constant $C_{n}{}^{2}$ is given by:

(32) $$\beta_{I,S}{}^{2}\left(L\right)\;=\;4\sigma_{\chi }^{2}\;=\;2.25\;*\;k^{\frac{7}{6}}\;*\;\int_{0}^{L}C_{n}{}^{2}\;*\;{\left[\frac{z\left(L-z\right)}{L}\right]}^{\frac{5}{6}}dz\;$$

The atmospheric turbulence intensity profile can be inverted by the iterative method. The entire detection range 0~*L* km is evenly divided into several parts, and the corresponding length interval of each part is ∆L. Assuming that the refractive index structure constant $C_{n}{}^{2}$ in each segment is a constant, then the $C_{n}{}^{2}$ in each segment can be directly extracted from the integral equation. Here, taking Equation (32) as an example, firstly, integral the range from zero to $L_{1}$, the corresponding spherical wave scintillation index $\beta_{I,S}{}^{2}$, $C_{n1}{}^{2}$ within zero to $L_{1}$ can be obtained. Then, by iterating in turn, the curve of $C_{n}{}^{2}$ changing with the detection height under the vertical detection path can be obtained. Therefore, using the hierarchical iteration method, Equation (32) can be converted to Equation (33).

(33) $$\beta_{I,S}{}^{2}\left(L\right)\;=\;2.25\;*\;k^{\frac{7}{6}}\;*\;\left\{\begin{array}{c} \int_{0}^{L_{1}}C_{n_{1}}^{2}\;*\;{\left[\frac{z\left(L_{1}-z\right)}{L_{1}}\right]}^{\frac{5}{6}}dz+\int_{L_{1}}^{L_{2}}C_{n_{2}}^{2}\;*\;{\left[\frac{z\left(L_{2}-L_{1}-z\right)}{L_{2}-L_{1}}\right]}^{\frac{5}{6}}dz+\ldots \\ +\int_{L_{n-1}}^{L_{n}}C_{n_{n}}^{2}\;*\;{\left[\frac{z\left(L_{n}-L_{n-1}-z\right)}{L_{n}-L_{n-1}}\right]}^{\frac{5}{6}}dz \end{array}\right\}$$

## 3. Simulation Results and Analysis

### 3.1. Comparison of Low-Frequency Sampling Optimization Results

**Figure 7:**
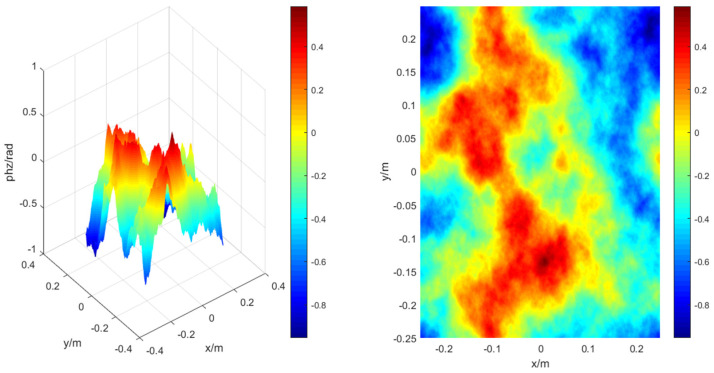
The three-dimensional (**left**) and two-dimensional (**right**) schematic diagrams of the phase distribution of the high-frequency phase screen numerically simulated in the turbulent atmosphere.

In the simulation model, the transmission of laser light in atmospheric turbulence is regarded as a series of phase screens placed in the Gaussian beam, including high-frequency phase screens and low-frequency compensated phase screens. Assuming that the turbulent spectrum structure is the Kolomogorov spectrum, the corresponding phase screen is constructed by the Fourier transform method, here, $C_{n}{}^{2}=2\times 10^{-15}$, *L* = 0.5 m, N×N=256×256, ∆z=200 m, and the transmission distance L=2000 m. Figure 7 shows the phase distribution of the high-frequency phase screen numerically simulated in the turbulent atmosphere.

**Figure 8:**
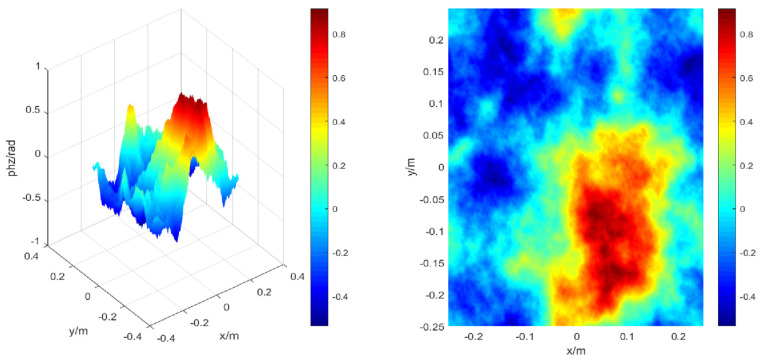
The three-dimensional (**left**) and two-dimensional (**right**) schematic diagrams of the numerically simulated phase distribution in the turbulent atmosphere after the third harmonic compensation under the same conditions.

In view of the shortcomings of the lack of low-frequency information in the phase screen constructed by the Fourier transform method, the low-order harmonic compensation is performed. According to the steps mentioned above, the schematic diagram of low-frequency compensation for numerical simulation of the phase distribution in a turbulent atmosphere is shown in Figure 8. It can be seen that the transition of the phase distribution is smoother.

**Figure 9:**
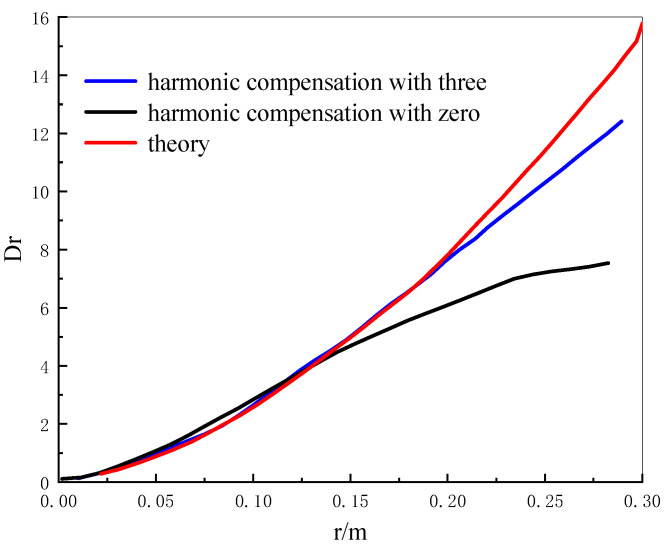
Kolmogorov phase screen structure function comparison diagram.

Figure 9 shows the comparison of the Kolmogorov phase screen structure function. It can be seen that the phase distribution obtained by the spectral inversion method is consistent in the high-frequency part, but obviously insufficient in the low-frequency part. However, the phase distribution after the third harmonic compensation is significantly improved in the low-frequency part.

### 3.2. Result Verification of Turbulent Phase Screen Simulation Model

**Figure 10:**
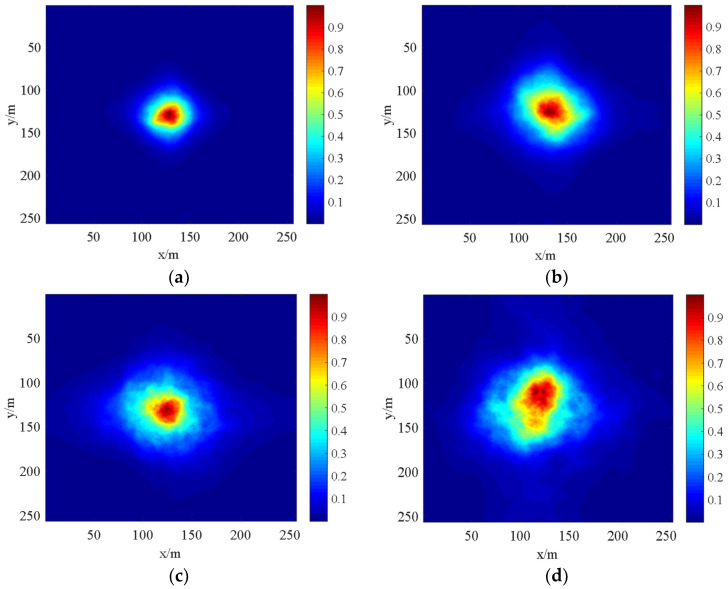
Light intensity distribution of the laser beam on the vertical path (**a**) Δz=75 m, L=1000 m, (**b**) Δz=75 m, L=2000 m, (**c**), Δz=200 m, L=1000 m (**d**) Δz=200 m, L=2000 m.

In order to test the reliability of the results of the turbulent phase screen simulation model, the scintillation index is chosen as the physical quantity to test this method. In order to simulate the transmission of the laser beam in atmospheric turbulence, we select the Gaussian distribution of the laser beam to pass through the phase screen shown in Figure 8. Figure 10 shows the light intensity distribution of the laser beam passing through the atmospheric turbulence on the vertical path. It can be seen that the phase disturbance generated by the Gaussian beam after passing through the turbulent phase screen makes the light intensity distribution no longer satisfy the Gaussian distribution. When the distance between phase screens (Δ*z*) remains the same, the discrete degree of the spot increases with the increase in the transmission distance (*L*); when the transmission distance (*L*) remains the same, the discrete degree of the spot becomes larger with the increase in the distance between the phase screen (Δ*z*).

When the conditions are $C_{n}^{2}=2\times 10^{-15}$, L=0.5 m and N×N=256×256, after changing the distance between the phase screens (Δ*z*) and the transmission distance (*L*), respectively, the light intensity distribution of the laser beam passing through the phase screen are shown as (a), (b), (c) and (d).

**Figure 11:**
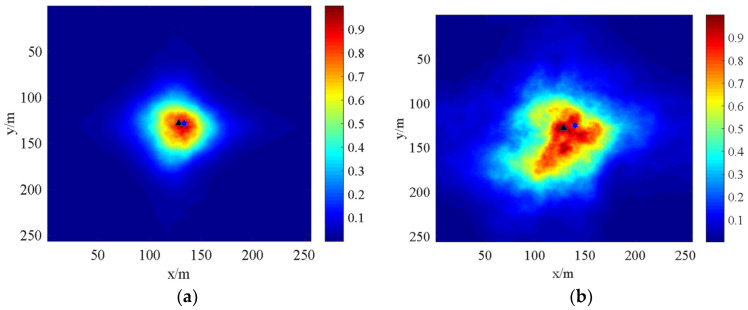
Spot drift phenomenon of laser transmission (**a**) Δz=75 m, L=2000 m, (**b**) Δz=200 m, L=2000 m.

For the light intensity distribution of the laser transmission on the vertical path, the spot drift phenomenon occurs. Figure 11 shows the spot drift phenomenon. In Figure 11, the hexagon indicates the original spot centroid located in the middle, while the triangle shows the centroid of the drifted spot. It is clear that the larger the value of the phase screen spacing Δ*z* is, the greater the discrete degree of the spot and the more obvious the phenomenon of light spot drifts.

When the conditions are $C_{n}^{2}=2\times 10^{-15}$,L=0.5 m and N×N=256×256, the distance between the phase screens (Δ*z*) and the transmission distance (*L*) are changed, respectively, and the spot drift of the laser beam passing through the phase screen are shown in (a) and (b).

**Figure 12:**
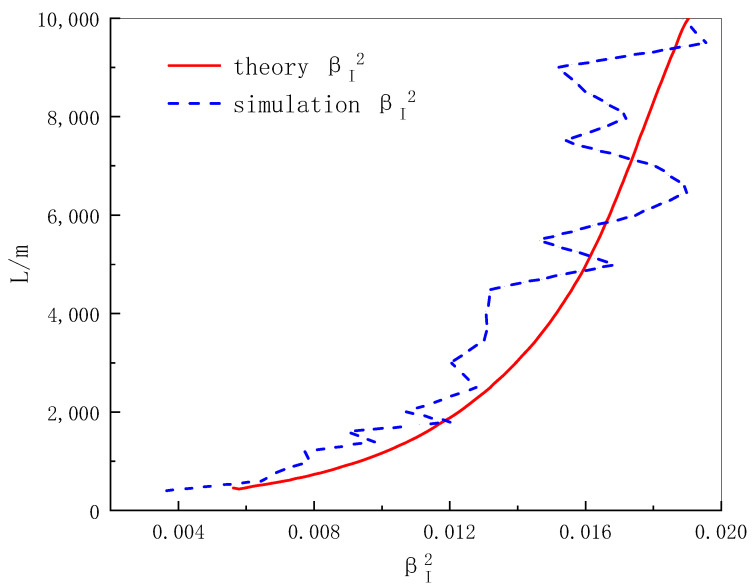
Distribution of scintillation index with transmission distance.

In this paper, the scintillation index of different heights is simulated and 10 spot images of the simulation results for each height are employed to obtain the corresponding scintillation index from the definition formula for each spot image. The above results are statistically averaged to obtain the scintillation index simulation value representing each height, and then compared with the Rytov theoretical results as shown in Figure 12. It can be seen that the change trend of the theoretical value and the simulation value is basically consistent.

### 3.3. Atmospheric Turbulence Profile

The Hufnagel-Valley 5/7(HV_5/7_) model is a common statistical model representing the turbulence profile in the mid-latitude inland region, and the expression is as follows [35]:

(34) $$C_{n}{}^{2}(h)=5.94\cdot 10^{-53}\cdot {\left(\frac{21}{27}\right)}^{2}\cdot h^{10}\cdot e^{\frac{-h}{1000}}+2.7\cdot 10^{-16}\cdot e^{\frac{-h}{1500}}+1.7\cdot 10^{-14}\cdot e^{\frac{-h}{100}}$$

**Figure 13:**
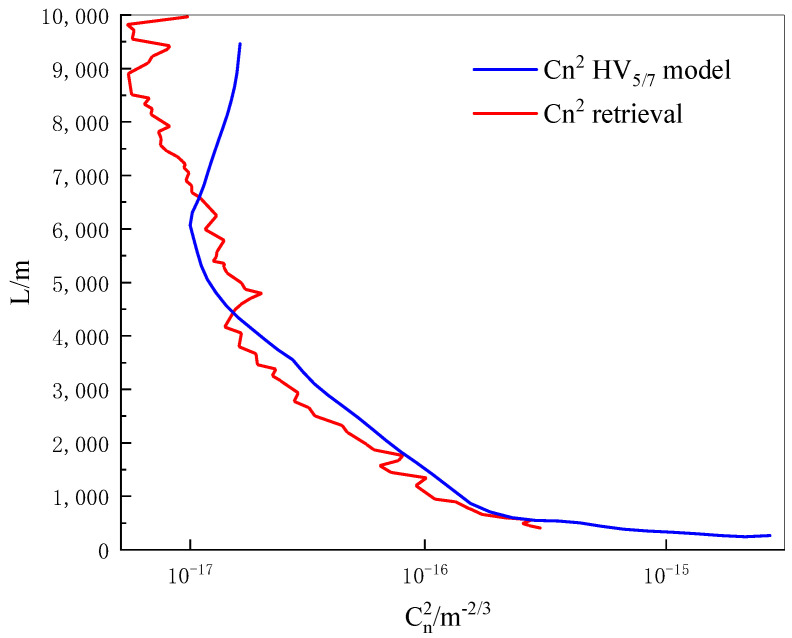
The profile of atmospheric refractive index structure constant obtained by the iterative inversion algorithm.

Figure 13 shows the profile of the atmospheric refractive index structure constant obtained by the iterative inversion algorithm. Compared with the input HV_5/7_ model, it can be seen that below the detection height of 4500 m, the $C_{n}{}^{2}$ obtained by inversion is slightly smaller than that of the HV_5/7_ model. When the detection height is 4500~6500 m, the $C_{n}{}^{2}$ inverted is slightly larger than that of the HV_5/7_ model. Therefore, the atmospheric refractive index structure constant profile obtained by the inversion algorithm is consistent with the input model below 6500 m, which preliminarily verified that the Mie-scattering lidar improved by the RTS theory has certain reliability in detecting atmospheric turbulence.

## 4. Conclusions

Numerical simulation is the most commonly used method to study atmospheric turbulence, and it can be used to verify and evaluate the atmospheric turbulence detection capability of lidar systems. The Fourier transform method is one of the methods in numerical simulation. In this paper, using RTS theory combined with a Mie-scattering lidar system in the laboratory, a phase screen simulation experiment for detecting atmospheric turbulence was designed. The Gaussian laser beam transmission in atmospheric turbulence was simulated by the Fourier transform method with the addition of the third harmonic, and a series of phase screens were used to simulate the phase disturbance and light intensity scintillation caused by atmospheric turbulence. The scintillation index can be calculated from the light intensity distribution of the phase screen. From the relationship between the scintillation index and the atmospheric refractive index structure constant in the vertical direction, the simulated atmospheric turbulence profiles are obtained by iterative inversion, and the accuracy of the phase screen construction is tested by the Rytov theory. The simulation results are shown as follows:

(1) Compared with the traditional Fourier transform method, the Fourier transform method with the addition of the third harmonic makes the phase distribution of the phase screen smoother, and it makes up for the shortcomings of the traditional method of insufficient low-frequency distribution;
(2) Comparing the spot scintillation index of Gaussian beam obtained by the Rytov theory, the trend of the scintillation index obtained by simulation is basically consistent with the theoretical value. The scintillation index gradually increases with the increase in the measurement distance, and it can be seen that the constructed phase screen is accurate;
(3) Comparing the atmospheric turbulence profile of the HV5/7 model, the atmospheric refractive index structure constant profile obtained by iterative inversion is in good agreement with the HV5/7 model below 6500 m; it fluctuates with the increase in the detection distance, generally increases and tends to be stable.

This study preliminarily verifies the reliability of the Mie-scattering lidar system for detecting atmospheric turbulence from a theoretical point of view, and provides a low-cost and easy-to-operate measurement method for detecting atmospheric turbulence in the future. In fact, the simulation of atmospheric turbulence has practical applications, such as the determination of the height of the top of the atmospheric boundary layer and the study of the wind load characteristics of the atmospheric boundary layer turbulence on low-rise buildings. At the same time, it provides a feasible research method for in-depth understanding of the direct and indirect effects of aerosols. Further research on the inversion algorithm is required in the later stage.

## Figures and Tables

**Table 1:** The system parameters of the Mie-scattering lidar.

Transmission System Parameter Information			
Laser	Semiconductor pumping (Nd:YAG)	Telescope	Schmidt–Cassegreen
Frequency	10 Hz	Diameter	10 inches
Pulse energy (355 nm, 532 nm, 1064 nm)	60–300 mJ	Laser Power Stability (532 nm)	≤2%
Pulse width (532 nm)	≤6 ns	Distance resolution	3 m
power	500 W	Control interface type	RS232
Receive system parameter information			
Focal length	2500 mm	Focal ratio	f/10
Spot stability	≤50 μrad	Spot diameter (1064 nm)	7 mm
PMT	Hamasatmu R3896	Field of view	0.4 mrad

## Data Availability

The aerosol particles size distribution and concentration data used to support the findings of this study are available from the corresponding author upon request.
